# Supplementary figures and images for: The therapeutically actionable long non-coding RNA ‘T-RECS’ is essential to cancer cells’ survival in NRAS/MAPK-driven melanoma
Source: Mol Cancer. 2024 Feb 22;23:40. doi: 10.1186/s12943-024-01955-7 (PMC10882889; doi:10.1186/s12943-024-01955-7)

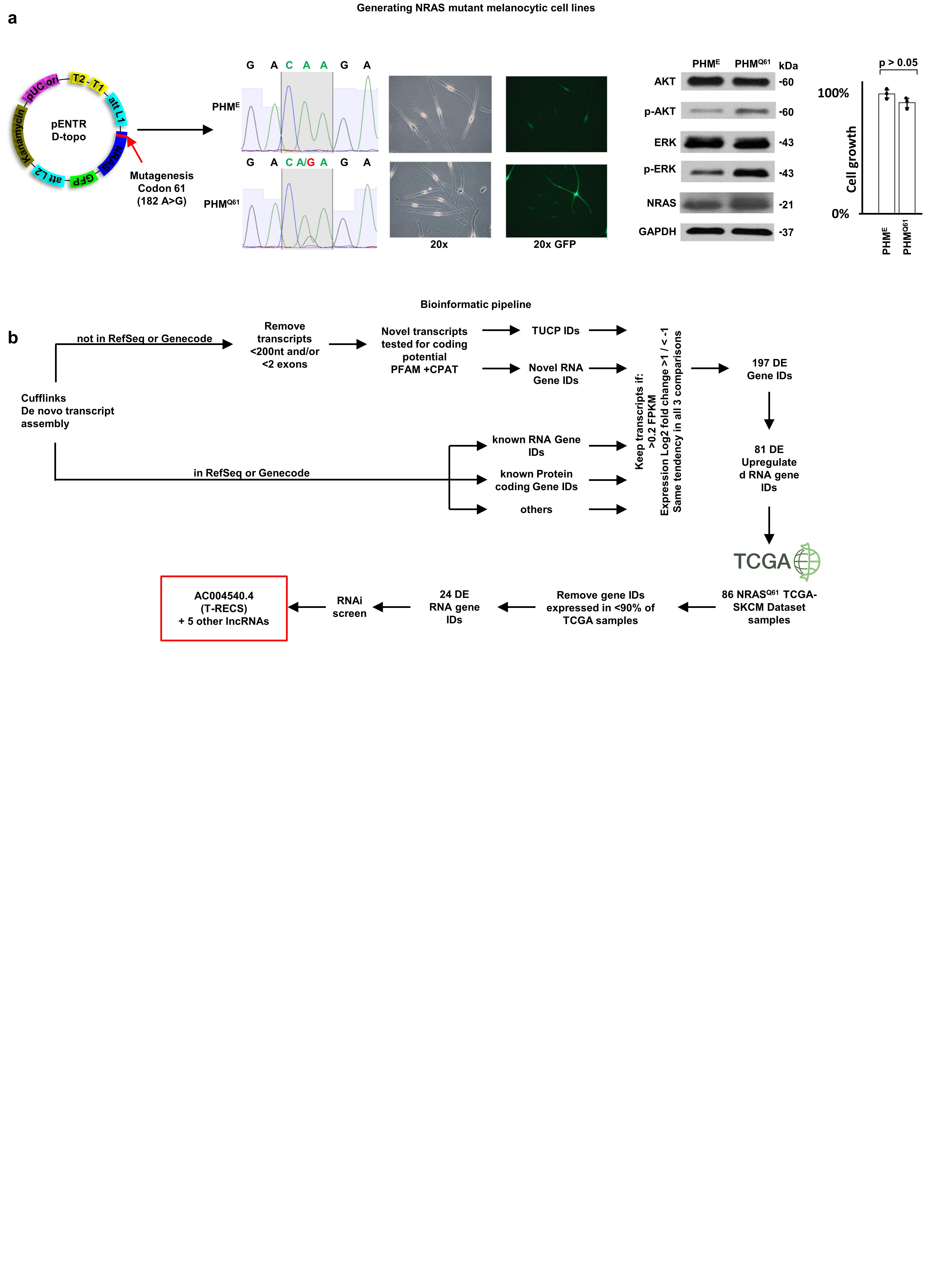

Supplement: Supplementary file 2 — Supplementary Fig. 1. Supplementary data of pipeline steps to identify lncRNAs that are MAPK-pathway associated. a) From left to right, a visual representation of the pENTR/D-TOPO plasmid that was used to create an NRAS mutant melanocytic cell-line (PHMQ61), including the rrnB_T2 and rrnB_T1 terminator sequences, the attL1 and attL2 recombination sites, the green fluorescent protein sequence (GFP), the kanamycin resistance gene, the pUC origin of replication sequence and the NRAS gene harboring a Q61 mutation in Codon 61. Sanger sequencing verified the successful insertion of an NRASQ61 mutation in PHMQ61, but not in melanocytic cell lines that were transfected with an empty vector (PHME). Microscopic imaging of PHMQ61 and PHME with co-expressed green fluorescent protein, which was used as a transduction efficacy reporter, is also shown. Immunoblotting shows induced MAPK signaling in form of upregulation of NRAS an the NRAS downstream signaling effectors p-AKT and p-ERK in PHMQ61 compared to PHME cells. GAPDH served as a loading control. PHME and PHMQ61 showed no significant differences in cell proliferation. ATP quantitation was used as marker for metabolically active cells and measured five days after seeding an equal number of cells (n = 3). P-value was calculated using Student’s t-test. b) The flowchart illustrates the individual bioinformatic pipeline steps for comparing and analyzing RNA-Seq results to identify T-RECS and five additional lncRNAs that are responsive to MAPK upregulation and essential for melanoma cell survival. The steps included the integration of data of 86 NRAS mutant patient-derived tumor samples from The Cancer Genome Atlas (TCGA) to filter for clinically relevant targets, and esiRNA screening to filter for their relevant role for cell survival. [file 12943_2024_1955_MOESM2_ESM.jpg]

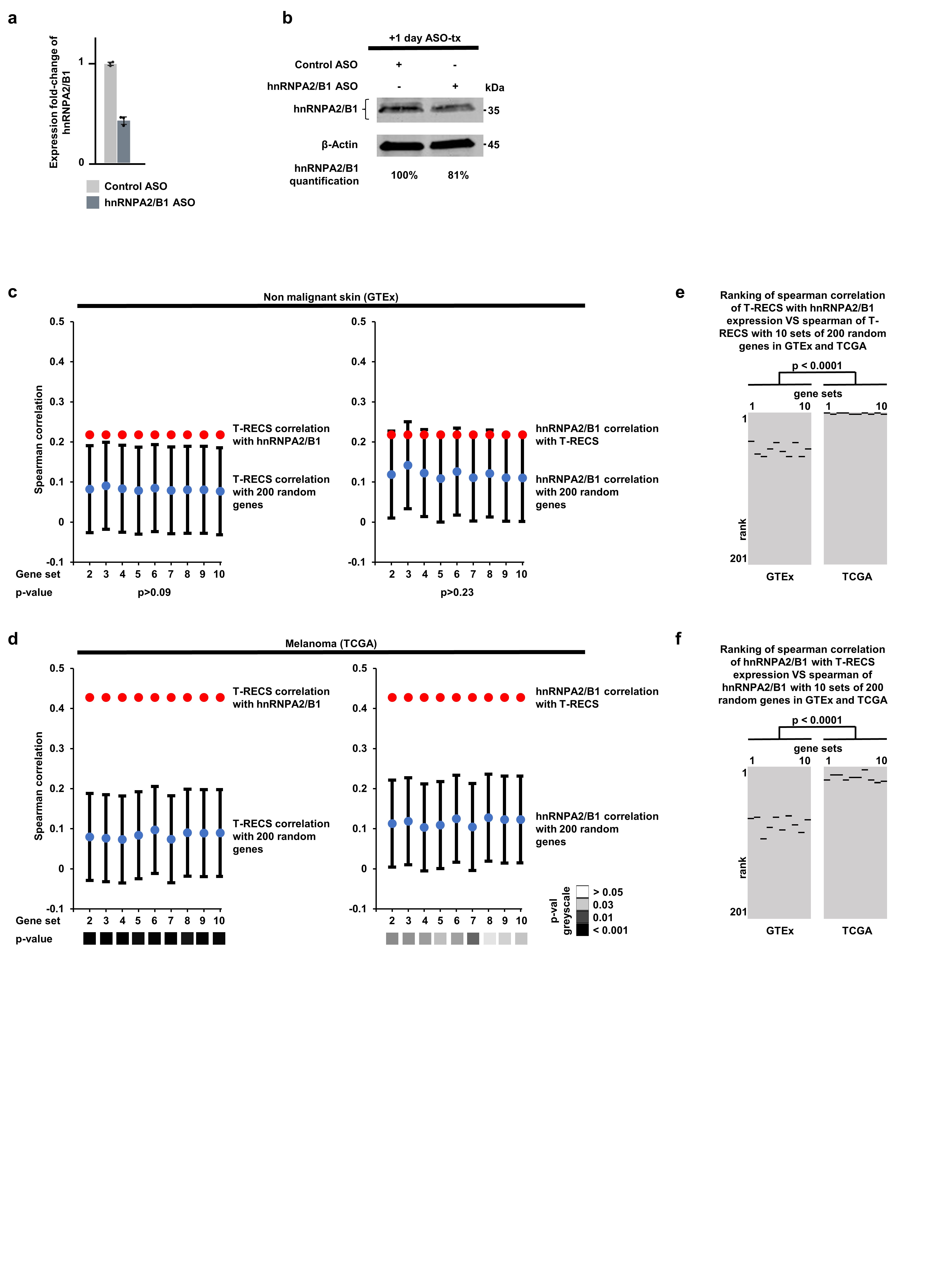

Supplement: Supplementary file 3 — Supplementary Fig. 2. Supplementary data of AC004540.4 (T-RECS) expression. a) List of the species conservation and coding probability scores for AC004540.4 (T-RECS) Ensembl Transcript IDs ENST00000451264 and ENST00000451368, as annotated in LNCipedia. T-RECS is not conserved in other species and identified as non-coding transcript by five different informatic tools. b) T-RECS expression analysis using, showing The lncRNA T-RECS is expressed in the tissue types heart and vessels (n = 2196), whole blood (n = 755), spleen (n = 241), lung (n = 578), GI tract (n = 2932), liver (n = 226), pancreas (n = 328), breast (n = 459), female reproductive tract (n = 506), male reproductive tract (n = 606), kidney and bladder (n = 110), nervous tissue (n = 3544), adrenal and thyroid gland (n = 911) and muscle and connective tissue (n = 3015). RNAseq data are derived from the patient’s biospies database GTEx. The median expression is shown by the center line, the box represents the lower and upper quartiles, and the whiskers extend to the furthest value that is less than 1.5 times the interquartile range from the lower and upper quartiles. The mean expression is marked by an ‘X’. [file 12943_2024_1955_MOESM3_ESM.jpg]

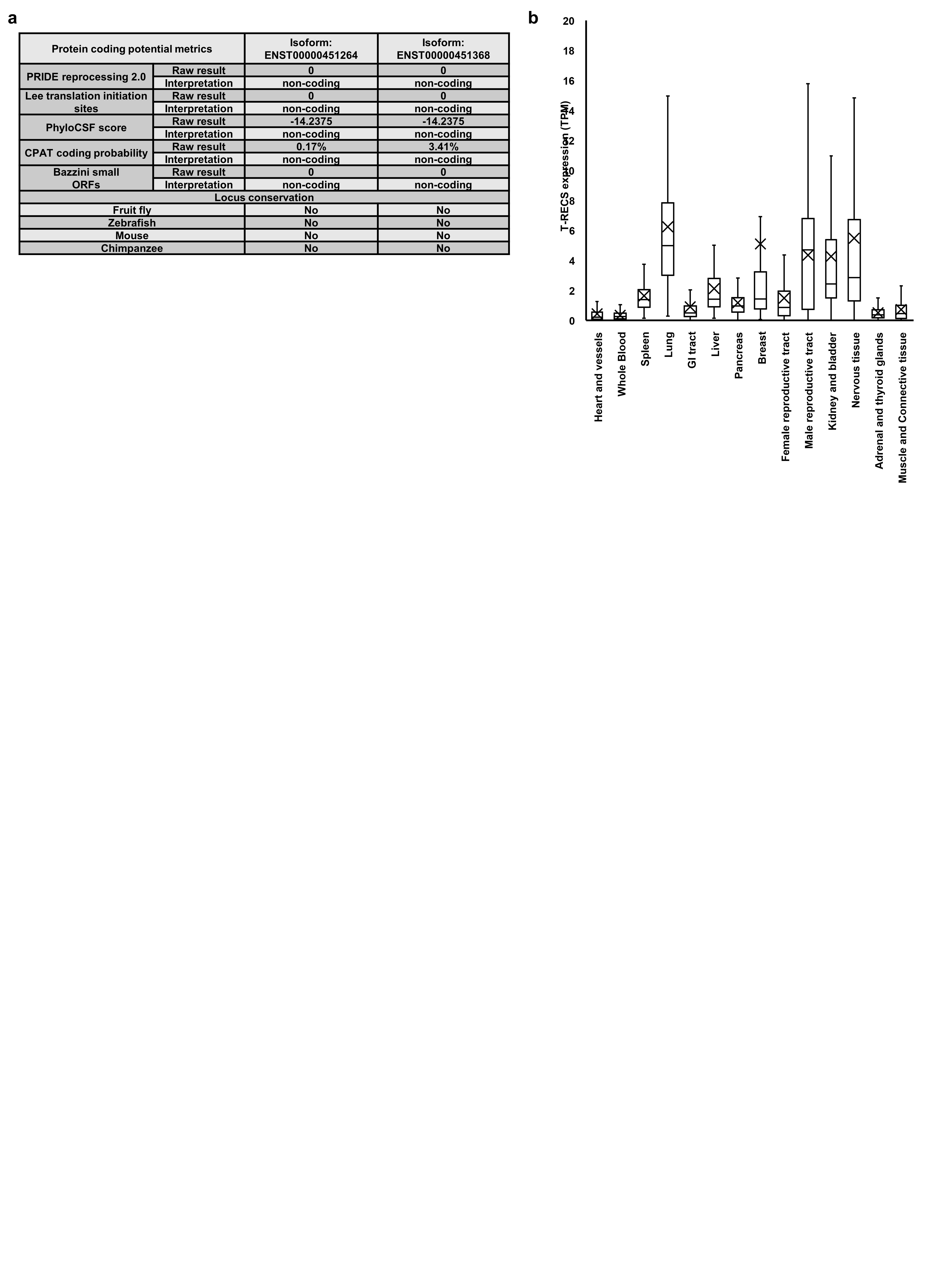

Supplement: Supplementary file 4 — Supplementary Fig. 3. Supplementary data of AC004540.4 (T-RECS) and hnRNPA2/B1 association. a) hnRNPA2/B1 ASO treatment specifically reduced hnRNPA2/B1 RNA levels, when compared to treatment with non-targeting Control ASO in the D04 cell line. b) Immunoblotting showing a limited decrease in hnRNPA2/B1 protein levels 1-day after hnRNPA2/B1 ASO treatment compared to Control ASO treatment in D04 cell lysate. β-actin served as a loading control. c-d) Extended analysis from Fig. 5i-j. The expression correlation coefficient of T-RECS and hnRNPA2/B1 is not significantly different from the coefficient of each of both genes compared to 9 sets of 200 randomly chosen genes in the GTEx database of healthy skin samples (GTEx, n = 1305). The comparisons of coefficients are significantly different in the TCGA dataset of MAPK-driven melanoma (n = 366). Significance was evaluated by comparing the correlation between T-RECS and hnRNPA2/B1 with correlations between either T-RECS or hnRNPA2/B1 with 200 randomly selected genes by calculating a Z-score. Error bars represent standard deviation. e-f) Ranking of the coefficient of expression correlation of T-RECS with hnRNPA2/B1 (black bars) and the correlation coefficients of either T-RECS (e) or hnRNPA2/B1 (f) with each gene of the 10 sets of 200 randomly chosen genes (grey bars) in TCGA and GTEx. The average ranking of the correlation of T-RECS with hnRNPA2/B1 significantly higher in TCGA compared to GTEx (T-RECS in GTEx: 29.8, in TCGA: 1.5, p < 0.0001; hnRNPA2/B1 in GTEx: 45.4, in TCGA: 9.3, p < 0.0001). [file 12943_2024_1955_MOESM4_ESM.jpg]
